# Molecular Cloning and Functional Studies of Two Kazal-Type Serine Protease Inhibitors Specifically Expressed by *Nasonia vitripennis* Venom Apparatus

**DOI:** 10.3390/toxins7082888

**Published:** 2015-08-04

**Authors:** Cen Qian, Qi Fang, Lei Wang, Gong-Yin Ye

**Affiliations:** 1 College of Life Science, Anhui Agricultural University, Hefei 230036, China; E-Mails: qiancenqiancen@163.com (C.Q.); wanglei20041225@163.com (L.W.); 2 State Key Laboratory of Rice Biology, Key Laboratory of Agricultural Entomology, Institute of Insect Sciences, Zhejiang University, Hangzhou 310058, China; E-Mail: fangqi@zju.edu.cn

**Keywords:** *Nasonia vitripennis*, Kazal-type, serine protease inhibitors, humoral immunity

## Abstract

Two cDNA sequences of Kazal-type serine protease inhibitors (KSPIs) in *Nasonia vitripennis*, *NvKSPI-1* and *NvKSPI-2*, were characterized and their open reading frames (ORFs) were 198 and 264 bp, respectively. Both *NvKSPI-1* and *NvKSPI-2* contained a typical Kazal-type domain. Real-time quantitative PCR (RT-qPCR) results revealed that *NvKSPI-1* and *NvKSPI-2* mRNAs were mostly detected specifically in the venom apparatus, while they were expressed at lower levels in the ovary and much lower levels in other tissues tested. In the venom apparatus, both *NvKSPI-1* and *NvKSPI-2* transcripts were highly expressed on the fourth day post eclosion and then declined gradually. The *NvKSPI-1* and *NvKSPI-2* genes were recombinantly expressed utilizing a pGEX-4T-2 vector, and the recombinant products fused with glutathione S-transferase were purified. Inhibition of recombinant GST-*NvKSPI-1* and GST-*NvKSPI-2* to three serine protease inhibitors (trypsin, chymotrypsin, and proteinase K) were tested and results showed that only *NvKSPI-1* could inhibit the activity of trypsin. Meanwhile, we evaluated the influence of the recombinant GST-*NvKSPI-1* and GST-*NvKSPI-2* on the phenoloxidase (PO) activity and prophenoloxidase (PPO) activation of hemolymph from a host pupa, *Musca domestica*. Results showed PPO activation in host hemolymph was inhibited by both recombinant proteins; however, there was no significant inhibition on the PO activity. Our results suggested that *NvKSPI-1* and *NvKSPI-2* could inhibit PPO activation in host hemolymph and trypsin activity *in vitro*.

## 1. Introduction

The Kazal-type serine protease inhibitors (KSPIs) comprise a large family of protease inhibitors. They are present widely in mammals, birds, crayfish, and insects and are named in reference to the work on the pancreatic secretory trypsin inhibitor first isolated by Kazal *et al*. [1]. During the 1950s–1980s, KSPIs were explosively studied in vertebrates, particularly mammals and birds [2]. Studies on KSPIs from invertebrates began in the 1990s when Friedrich *et al*. first reported a double-headed Kazal-type thrombin inhibitor, rhodniin, from *Rhodnius prolixus* (Hemiptera: Reduviidae) [3]. In 1994, a four-domain Kazal protease inhibitor from the blood cells of crayfish *Pacifastacus leniusculus* (Crustacea: Decapoda) [4] and a leech-derived tryptase inhibitor from the medicinal leech *Hirudo medicinalis* (Hirudinea: Hirudinidae) [5] were documented, respectively. After that, studies on invertebrate KSPIs have extended to other invertebrate species including shrimp, blood-sucking insects, silk moths, locusts, and so on [6,7,8,9,10,11,12].

KSPIs have the conserved structures of one or more Kazal domains (KDs). A typical KD is composed of 40–60 amino acids including six cysteine residues and the following conservative motif: C1-X(1-7)-C2-X(5)-PVC3-X(4)-TYXNXC4-X(2-6)-C5-X(9-16)-C6. These six cysteine residues formed three intra-domain disulfide bridges between cysteine numbers 1–5, 2–4, and 3–6, resulting in a characteristic three-dimensional structure [13]. So far, hundreds of KSPIs with various functions have been reported [14]. KSPIs are involved in many important physiological processes, such as embryogenesis, development, excessive autophagy, microbial invasion, inflammation, and immune responses [15,16,17,18,19]. Native KSPIs from blood-feeding arthropods can inhibit trypsin, thrombin, elastase, chymotrypsin, plasmin, subtilisin A, and factor XIIa [3,20,21,22,23].

Parasitic wasps are natural resources that play an important role in biological control. They lay eggs into hosts or on the surface of hosts along with maternal and embryonic factors such as venom, polydnavirus (PDV), virus-like particles (VLP), ovarian proteins, teratocytes, and proteins secreted from larvae to interfere with the host immune responses for successful parasitization [24,25,26]. Unlike ichneumonid and braconid parasitoids, *Nasonia vitripennis* (Hymenoptera: Pteromalidae) injects venom, but not PDVs, into its host after oviposition. *N. vitripennis* venom is responsible for multiple functions in regulating the physiological processes of its host including induction of pathological and ultrastructural changes in cultured cells, interfering with the cellular immunity of host hemocytes, causing cell death, stimulation of intracellular calcium release in cultured cells, and disruption of the pupariation and eclosion behavior of the host [27,28,29]. Using proteomics methods, De Graaf *et al.* [30] previously identified 79 venom proteins from *N. vitripennis*, two of which are KSPIs (NCBI accession numbers NM_001161523 and NM_001170879). Recently, our group determined the transcriptome and proteome of venom gland and other residual tissues in *N. vitripennis* by RNA-seq (RNA sequencing) and LC-MS/MS (liquid chromatography–tandem mass spectrometry) methods [31], and we also detected two KSPIs in venom, as described by de Graaf *et al.* [30].

*N. vitripennis* is not only an advantageous ectoparasitoid to flies but an ideal perfect model insect for genetic and developmental biology studies [32,33]. In addition, genome sequences and comparative analyses have been reported for *N. vitripennis* and two other closely related parasitoid wasps, *N. giraulti*, and *N. longicornis* (Hymenoptera: Pteromalidae) [34]. While these reports document progress in understanding the composition of *N. vitripennis* venom, information on the activity and functional molecular mechanisms of venom actions are still lacking.

Here, we molecularly characterized two KSPIs (*NvKSPI-1* and *NvKSPI-2*) in *N. vitripennis* and determined their tissue and developmental expression patterns. We also tested the inhibition of recombinant *NvKSPI-1* and *NvKSPI-2* on three serine protease inhibitors’ *in vitro* and PO activity and PPO activation of host hemocytes. These results will provide further insight into the role of KSPIs in insects, especially in parasitoid wasps.

## 2. Results

### 2.1. Molecular Characterization of NvKSPI-1 and NvKSPI-2

**Figure 1:**
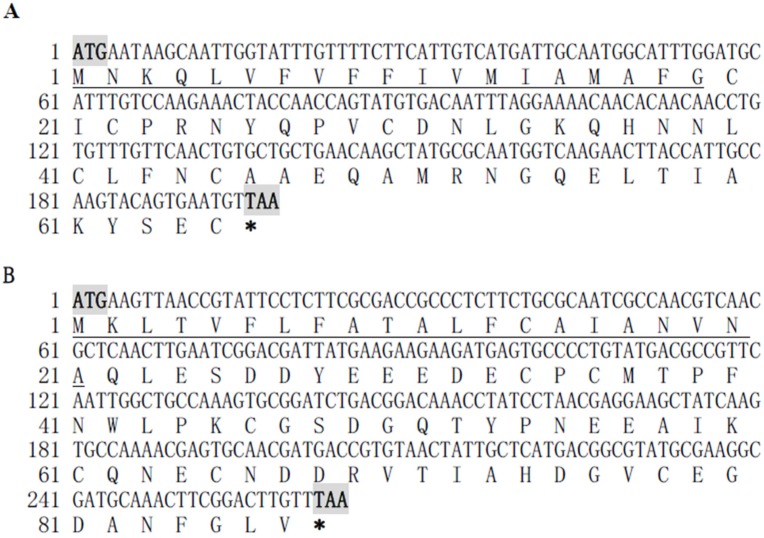
Nucleotide and deduced amino sequences of *NvKSPI-1* (**A**) and *NvKSPI-2* (**B**) cDNAs from *N. vitripennis*. Signal peptides are underlined; the initiator codon “ATG” and terminator codon “TAA” are bolded and highlighted.

**Figure 2:**
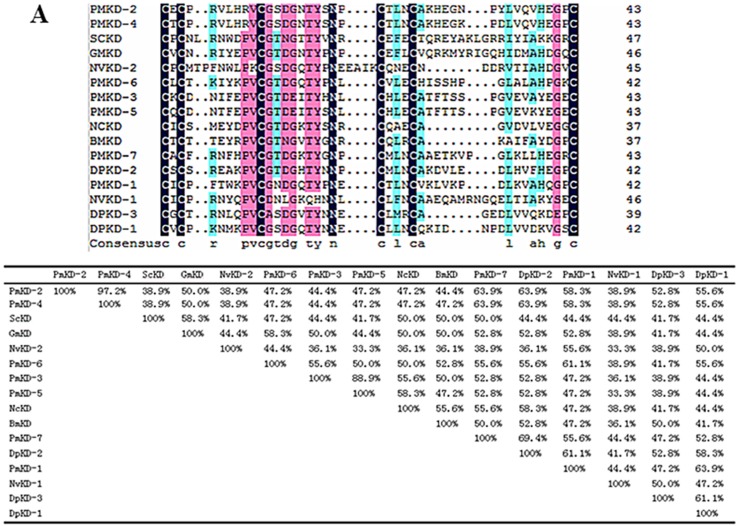

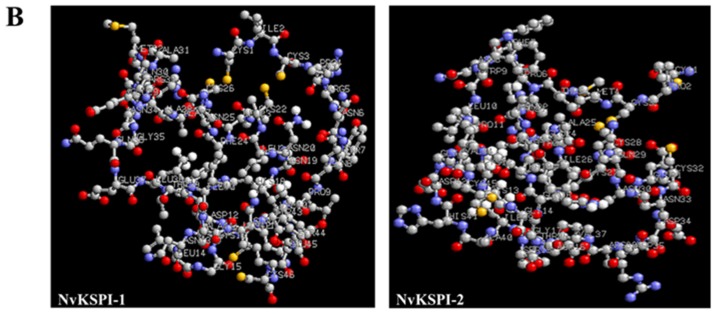
Sequence analysis of *NvKSPI-1* and *NvKSPI-2*. (**A**) Multiple sequences alignment and amino acid identity analysis of Kazal domains (KDs) between *NvKSPI-1*, *NvKSPI-2*, and other KSPIs. (**B**) Predicted tertiary structure of *NvKSPI-1* and *NvKSPI-2* KDs by phyre^2^ on line. Yellow spheres represent cysteines forming three intra-domain disulfide bridges between cysteine numbers 1–5, 2–4, and 3–6. The corresponding species of abbreviations and their GenBank accession numbers are as follows: PMKD-1,2,3,4,5,6,7: *Panstrongylus megistuss* (ADF97836); SCKD: *Stomoxys calcitrans* (AAY98015); GMKD: *Glossina morsitans* (AFG28187); NCKD: *Neospora caninum* (AAM29188); BMKD: *Bombyx mori* (NP_001037047); DPKD-1,2,3: *Danaus plexippus* (EHJ76238); NVKVD1 and NVKVD2: *Nasonia vitripennis* (NM_001161523 and NM_001170879).

**Figure 3:**
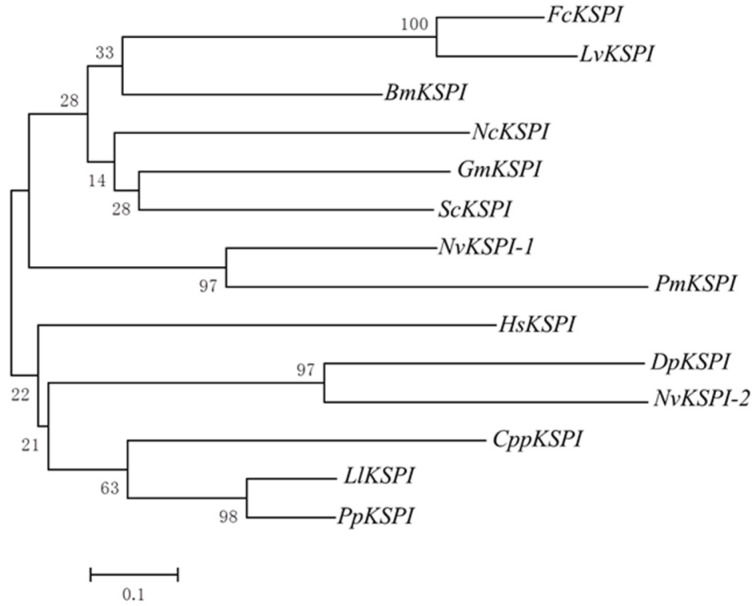
Phylogenetic analysis of *NvKSPI-1*, *NvKSPI-2*, and other KSPIs’ amino acid sequences based on the neighbor-joining method. The origin of amino acid sequences and their GenBank accession numbers are as follows: *FcKSPI*: *Fenneropenaeus chinensis* (ABC33915); *LvKSPI*: *Litopenaeus vannamei* (AAT09421); *BmKSPI*: *Bombyx mori* (NP_001037047); *NcKSPI*: *Neospora caninum* (AAM29188); *GmKSPI*: *Glossina morsitans* (AFG28187); *ScKSPI*: *Stomoxys calcitrans* (AAY98015); *PmKSPI*: *Panstrongylus megistus* (ADF97836); *HsKSPI*: *Homo sapiens* (NP_115955); *DpKSPI*: *Danaus plexippus* (EHJ76238); *CppKSPI*: *Culex pipiens pallens* (AFN41343); *LlKSPI*: *Lutzomyia longipalpis* (ABV60319); *PpKSPI*: *Phlebotomus papatasi* (ABV44739); *NvKSPI-1* and *NvKSPI-2*: *Nasonia vitripennis* (NM_001161523 and NM_001170879).

Fragments of *NvKSPI-1* and *NvKSPI-2* containing 198 and 264 nucleotides were amplified and sequenced; they respectively encoded 65 and 87 amino acids with predicted secretory *N*-terminal signal peptides (Figure 1). BLASTn results showed that these two cDNA sequences were completely consistent with *N. vitripennis* KSPIs in the NCBI database (NM_001161523 and NM_001170879). A multiple sequence alignment of KDs from *NvKSPI-1*, *NvKSPI-2*, and other KSPIs showed that although the numbers of KDs in these species varied from one to seven, typical KD motifs were highly similar, including the six cysteine residues that formed disulfide bonds between cysteine numbers 1–5, 2–4, and 3–6 (Figure 2). Phylogenetic analyses of *NvKSPI-1* and *NvKSPI-2* with homologs in 12 other species using their mature peptide region sequences indicated that *NvKSPI-1* and *NvKSPI-2* were not classified into the same cluster and showed close genetic distances with *Danaus plexippus* (Lepidoptera: Danaidae) and *Panstrongylus megistus* (Hemiptera: Reduviidae), respectively (Figure 3). In Diptera insects, *Lutzomyia longipalpis* (Diptera: Psychodidae) and *Phlebotomus papatasi* (Diptera: Psychodidae) are classified into one cluster, while *Glossina morsitans* (Diptera, Glossinidae) and *Stomoxys calcitrans* (Diptera: Muscidae) are classified into another cluster. In general, *NvKSPI-1* and *NvKSPI-2* were genetically closer to insects, and farther from crustaceans and vertebrates.

### 2.2. Expression and Purification of Recombinant NvKSPI-1 and NvKSPI-2

**Figure 4:**
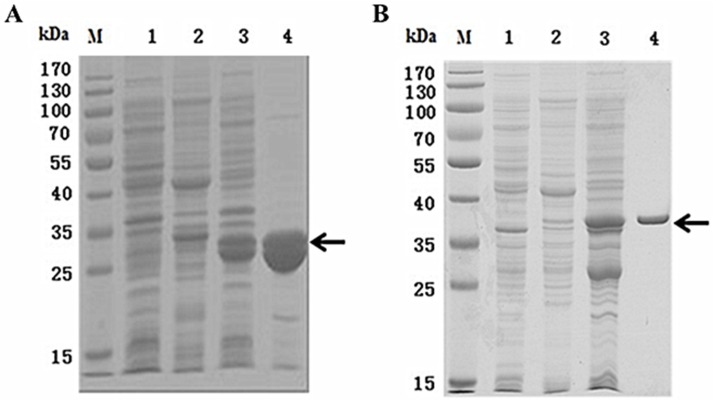
SDS-PAGE analysis of *NvKSPI-1* (**A**) and *NvKSPI-2* (**B**) recombinant proteins. M: Protein molecular weight marker; 1: Not induced by IPTG; 2: Precipitation after IPTG induction. 3: Supernatant after IPTG induction; 4: Purified proteins.

Two recombinant proteins fused with glutathione S-transferase at their *N*-terminuses, namely GST-*NvKSPI-1* and GST-*NvKSPI-2*, were successfully detected by sodium dodecyl sulfate polyacrylamide gel electrophoresis (SDS-PAGE) with molecular weights of about 33 kDa and 36 kDa, respectively. The recombinant proteins were mainly detected in the supernatant and not in the precipitate. As soluble fusion proteins, GST-*NvKSPI-1* and GST-*NvKSPI-2* were purified with the GST•Bind™ Resin Kit (Novagen, Hilden, Germany) and the purified proteins were analyzed by SDS-PAGE (Figure 4). The protein concentrations of purified GST-*NvKSPI-1* and GST-*NvKSPI-2* were 1.8 mg/mL and 2.1mg/mL, respectively, and the concentration of the GST fusion proteins was 2.4 mg/mL.

### 2.3. Transcriptional Profiles of NvKSPI-1 and NvKSPI-2 in Different Tissues and Developmental Stages

**Figure 5:**
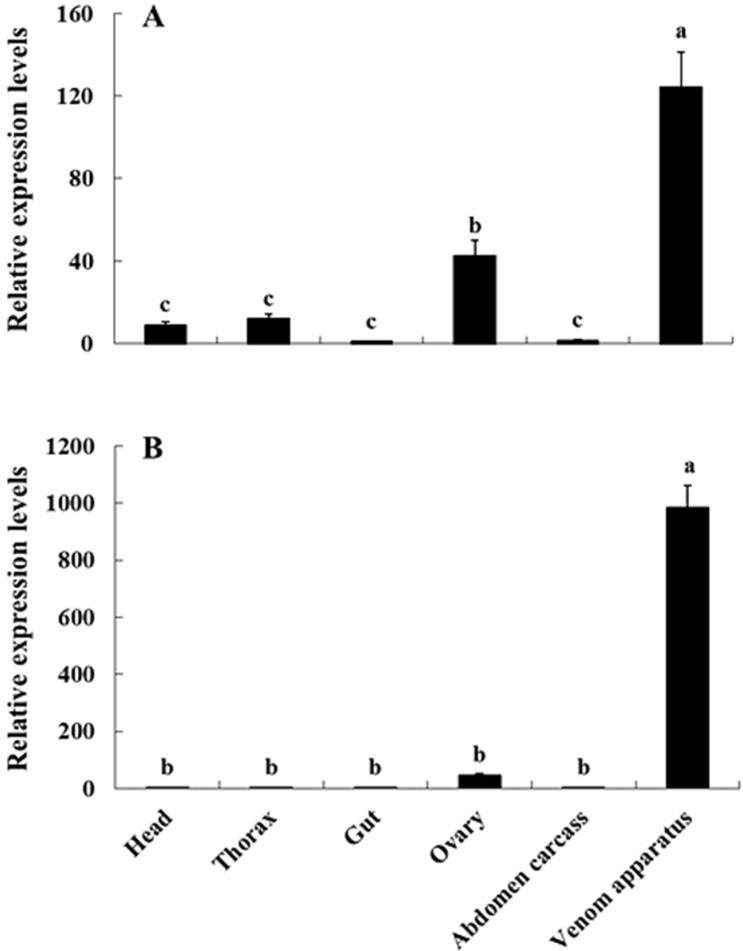
Tissue distribution of transcript levels of *NvKSPI-1* (**A**) and *NvKSPI-2* (**B**) in *N. vitripennis*. Abdomen carcass represents the rest of abdomen post dissection. Venom apparatus contains venom reservoir and gland, and venom released. All values in the figure are represented as mean ± standard deviation. Bars labeled with different letters are significantly different (one-way ANOVA followed by LSD test, *p* < 0.05).

**Figure 6:**
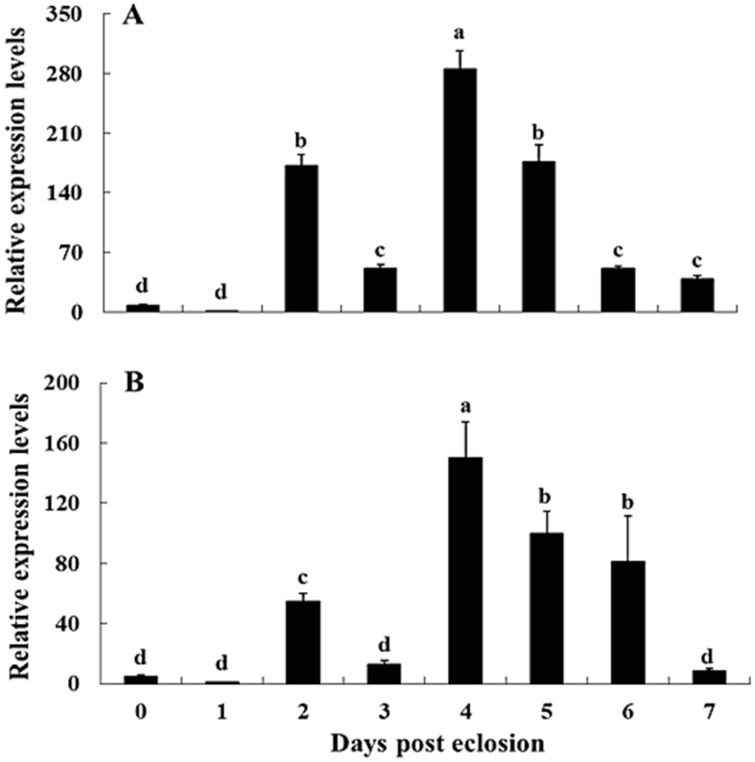
Developmental stages of transcript levels of *NvKSPI-1* (**A**) and *NvKSPI-2* (**B**) in venom apparatus of *N. vitripennis*. Female adults were sampled on days 0 to 7 post eclosion. All values are presented as mean ± standard deviation. Bars labeled with different letters are significantly different (one-way ANOVA followed by LSD test, *p* < 0.05).

Levels of *NvKSPI-1* and *NvKSPI-2*, confirmed by genes by RT-qPCR using *18S rRNA* as the reference gene, showed similar transcript profiles (Figure 5 and Figure 6). In tissues, *NvKSPI-1* and *NvKSPI-2* mRNAs were both expressed specifically in the venom apparatus, while they were detected at lower levels in the ovary and much lower amounts in other tissues tested. In detecting the transcript levels of *NvKSPI-1* and *NvKSPI-2* at different developmental stages (0–7 days post eclosion) in the venom apparatus of female adults, they were found to be lower at 0, 1 and 3 days post eclosion, but higher on the second day and highest on the fourth day post eclosion, after which they declined gradually. *NvKSPI-1* and *NvKSPI-2* had similar transcript profiles.

### 2.4. Serine Protease Inhibition Activity of NvKSPIs

**Figure 7:**
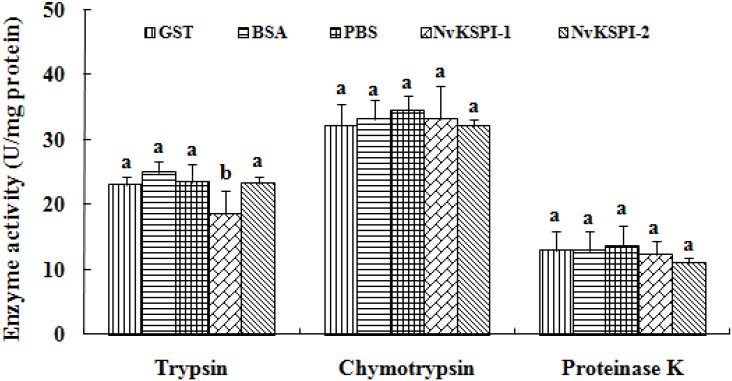
Effects of recombinant *NvKSPI-1* and *NvKSPI-2* on the activity of serine proteases. GST: GST-tag protein expressed by pGEX-4T-2; BSA and Buffer: treated by BSA and reaction buffer respectively; *NvKSPI-1* and *NvKSPI-2*: treated by recombination proteins respectively. All values in the figure are represented as mean ± standard deviation. Bars labeled with different letters are significantly different (one-way ANOVA followed by LSD test, *p* < 0.05).

To define whether *NvKSPI-1* and *NvKSPI-2* can affect serine protease activity, we determined their enzyme activity inhibition spectrum to trypsin, chymotrypsin, and proteinase K with recombinant GST-*NvKSPI-1* and GST-*NvKSPI-2*. Results showed that only *NvKSPI-1* could inhibit the activity of trypsin, while *NvKSPI-2* did not inhibit the activity of any of the three serine proteases tested (*F* = 4.159, *p* = 0.04; *F* = 0.247, *p* = 0.932; *F* = 0.552, *p* = 0.714, respectively) (Figure 7).

### 2.5. Effects of NvKSPIs on Prophenoloxidase (PPO) Activation and Phenoloxidase (PO) Activity

**Figure 8:**
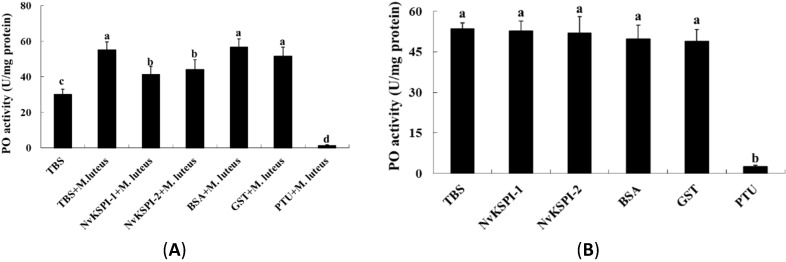
Effects of recombinant *NvKSPI-1* and *NvKSPI-2* on the PPO activation (**A**) and PO activity (**B**) of hemolymph of *M. domestica* pupae. (**A**) Screened hemolymph was incubated with TBS alone, or TBS/*M. luteus*, *NvKSPI-1*/*M. luteus*, *NvKSPI-2*/*M. luteus*, BSA/*M. luteus*, GST/*M. luteus*, PTU/*M. luteus* in TBS for 20 min at 25 °C. PPO activation was assayed using l-dopamine as a substrate, as described in Materials and Methods. (**B**) Screened hemolymph was incubated with *M. luteus* in TBS for 10 min at 25 °C, and then incubated with TBS, *NvKSPI-1*, *NvKSPI-2*, BSA, GST, or PTU for 10 min at 25 °C. PO activity was assayed using l-dopamine as a substrate, as described in Materials and Methods. All values in the figure are represented as mean ± standard deviation. Bars labeled with different letters are significantly different (one-way ANOVA followed by LSD test, *p* < 0.05).

To test whether *NvKSPI-1* and *NvKSPI-2* played a role in PPO activation and PO of hemolymph of host pupae, inhibition of PPO activation and PO activity were tested with recombinant GST-*NvKSPI-1* and GST-*NvKSPI-2* and compared with positive and negative controls. The PO activity between samples incubated with *NvKSPI-1*/*M. luteus* and *NvKSPI-2*/*M. luteus* were significantly different compared with the BSA and GST controls (*F* = 73.255, *p* < 0.001) (Figure 8A). The results indicated that recombinant GST-*NvKSPI-1* and GST-*NvKSPI-2* could inhibit PPO activation in the hemolymph of the host. When PPO in the hemolymph was pre-activated with *M. luteus*, recombinant GST-*NvKSPI-1* and GST-*NvKSPI-2* had no effect on PO activity (F = 27.26, *p* < 0.001) (Figure 8B). These results suggested that *NvKSPI-1* and *NvKSPI-2* could inhibit PPO activation in the host hemolymph but could not inhibit PO activity after PPO was activated.

## 3. Discussion

Structural studies of KSPIs have shown that they usually possess one to several conserved KDs consisting of six cysteine residues, but active sites of KSPIs have become highly variable due to long-term evolution [15]. Studies on *P. leniusculus* and *Penaeus monodon* (Crustacea: Decapoda) revealed that there were at least 26 and 20 different KDs from the hemocyte KSPIs of *P. leniusculus* and *P. monodon*, respectively. The position of the P1 site (usually the second amino acid residue after the second cysteine), a determinant for substrate specificity, varied highly [2,35]. In general, the domain with the P1 site of Arg or Lys inhibits trypsin; the P1 site of Tyr, Leu, Pro, Phe, or Met inhibits chymotrypsin; the P1 site of Ala or Ser inhibits elastase; and the P1 site of Thr or Asp inhibits proteinase K or subtilisin A.

Here, we characterized two KSPIs in *N. vitripennis* (*NvKSPI-1* and *NvKSPI-2*); both have a typical KD consisting of six cysteine residues. For *NvKSPI-1*, the P1 site is Arg, and it was supposed to inhibit the activity of trypsin but not chymotrypsin (or proteinase K). On the other hand, the P1 site of *NvKSPI-2* is Thr, and it was supposed not to inhibit the activity of our tested serine proteases (trypsin, chymotrypsin, and proteinase K). Our results with recombinant *NvKSPI-1* and *NvKSPI-2* were consistent with the general principle. As one of the most important families of protease inhibitors, KSPIs not on played a role in protecting the pancreas of vertebrates, but are also involved in many physiological processes in arthropods, such as dissolution, food digestion, blood coagulation, embryogenesis, ontogenesis, and inflammation and immune responses [16,36]. In two common shrimps, *Litopenaeus vannamei* (Crustacea: Decapoda) and *Fenneropenaeus chinensis* (Crustacea: Decapoda), KSPIs were cloned from their hemocytes’ cDNA libraries, and the mRNA levels of the shrimp KSPIs were upregulated after injection of a Gram-negative marine bacterium, *Vibrio alginolyticus*, suggesting a probable role of KSPIs in the immune response [7,9]. In the black tiger shrimp, *P. monodon*, a specific KSPI, SPIPm2, did not function as a protease inhibitor but inhibited the regulatory function of WSV477. Interaction between SPIPm2 and viral protein WSV477 reduced the replication of the white spot syndrome virus [10]. In bivalves, a five-domain KSPI was identified from the pearl oyster *Pinctada fucata* (Mollusca: Bivalvia) that could inhibit chymotrypsin and trypsin activities [37]. Two KSPIs (MdSPI-1 and MdSPI-2) that are important humoral factors of innate immunity were identified from the surf clam *Mesodesma donacium* (Mollusca: Bivalvia). They were significantly upregulated at 2 and 8 h post infection with *V. anguillarum* [38]. In the coconut rhinoceros beetle, *Oryctes rhinoceros* (Coleoptera: Scarabaeidae), a single-domain KSPI was shown to play a key role in protection against bacterial infections [39]. In blood-sucking bugs *Rhodnius prolixus* (Hemiptera: Reduviidae) and *Dipetalogaster maximus* (Hemiptera: Reduviidae), synthesized double-headed KSPIs, which are highly specific for thrombin, could prevent the host blood coagulation [3,40]. In *Triatoma infestans* (Hemiptera: Reduviidae), one KSPI, factor XIIa, was found to participate in its meal acquisition and digestion, while another KSPI, Infestin 1R, was also demonstrated to be able to impair mammalian cell invasion by *Trypanosoma cruzi* [21]. In the mosquito, *Aedes aegypti* (Diptera: Culicidae), KSPIs not only acted as anticoagulants during blood feeding and digestive processes, but served other functions during the development of the mosquito [41]. In silk moths, *Bombyx mori* (Lepidoptera: Bombycidae), one three-domain KSPI was identified from the cDNA library of the pupae, and was speculated to inhibit the invasion of pathogenic microorganisms [11]. In Asian honey bees, *Apis cerana* (Hymenoptera: Apidae), a venom KSPI acting as a microbial serine protease inhibitor was identified and demonstrated to have inhibitory activity against subtilisin A and proteinase K [42]. In the desert locust, *Schistocerca gregaria* (Orthoptera: Acrididae), one KSPI specific for elastase, subtilisin, and chymotrypsin were identified from its ovary gland [12]. Therefore, KSPIs have diverse functions in insects, particularly related to insect immune responses.

Compared to other insects, few studies on KSPIs have been reported in parasitoid wasps. Only de Graaf *et al.* [30] and our group identified two cDNA sequences of KSPIs (*NvKSPI-1* and *NvKSPI-2*) from *N. vitripennis*, and our previous proteome determination of venom of *N. vitripennis* indicated that *NvKSPI-1* and *NvKSPI-2* could be secreted into the venom (unpublished), but no further studies were performed. Parasitoid wasps are important potential resources for biological control. *N. vitripennis* is an ectoparasitoid of flies and deposits its eggs along with the venom to ensure the development of the offspring by inhibiting the immune response or growth and development of the host [43,44,45]. Here, we presented a detailed study on the functions of *NvKSPI-1* and *NvKSPI-2* to host immunity. Our results showed that *NvKSPI-1* and *NvKSPI-2* were both expressed in *N. vitripennis* venom apparatus, in which their transcribed levels were hundreds of times higher than in other tissues tested, suggesting that *NvKSPI-1* and *NvKSPI-2* were possibly involved in inhibition of the host immune response. For the expression profiles of *NvKSPIs* in venom apparatus, the expression level of *NvKSPIs* declined at 3 days post eclosion compared to that of the second day; this might be due to the biological rhythm of female wasps. The first three days post eclosion were the main mating and oviposition periods; after oviposition peak, female wasps would re-express *NvKSPIs* for the subsequent parasitism. Of course, *NvKSPIs* might be involved in other biological functions during the eclosion period. PPO and PO activation assays of hemolymph from *M. domestica* (host) pupae showed that *NvKSPI-1* and *NvKSPI-2* were inhibitors of PPO. The PPO activation system is an important component of the immune system in arthropods, and activation of zymogen PPO to active PO involves a serine protease cascade [46,47] and is tightly regulated by serine proteases and serpins [48,49,50]. *N. vitripennis*, as an important ectoparasitoid, had two KSPIs (*NvKSPI-1* and *NvKSPI-2*) that were expressed specifically in venom apparatus and secreted into venom, suggesting that *NvKSPI-1* and *NvKSPI-2* played a role in repressing host melaninization through inhibition of trypsin activity and PPO activation. However, more studies are needed to investigate how *NvKSPI-1* and *NvKSPI-2* inhibit PPO activation and screen for their potential interaction factors.

## 4. Materials and Methods

### 4.1. Insect Rearing

Cultures of *Musca domestica* (Diptera: Muscidae) and *N*. *vitripennis* were collected from the experimental field of Zhejiang University, Hangzhou, China. The laboratory colonies of *N*. *vitripennis* and its host *M*. *domestica* were maintained as described previously [51] and used in all experiments. In brief, the host larvae were fed an artificial diet composed of 15% milk powder, 35% wheat bran, and 50% water, in 500 mL glass canning jars (at 25 ± 1 °C, light:dark = 10h:14h, relative humidity (R. H.) = 75%) until eclosion. *M. domestica* adults were maintained on a mixture of sugar and milk powder (10:1) and water, within a stainless steel-mesh cage (55 cm × 55 cm × 55 cm) under the same conditions just described. Freshly pupated hosts were exposed to mated female wasps (pupae:wasps = 1:10) in a 500 mL glass canning jar for 24 h. The parasitized pupae were maintained under the conditions just described. After emerging, the female wasps were collected and held in glass containers (also under the conditions just described) and fed *ad lib* on 20% (*v/v*) honey solution to lengthen the life span for 3–4 days until dissection of the venom reservoir and gland.

### 4.2. Sample Preparation

Female *N. vitripennis* from 0 to 7 days post eclosion were collected and paralyzed for 5 min at −70 °C. The head, thorax, gut, ovary, remaining abdomen carcass (the rest of the abdomen after dissection), and venom apparatus (containing venom reservoir and gland) were then collected on ice under a stereoscope (Lecia, Frankfurt, Germany). Samples were stored at −70 °C.

### 4.3. RNA Extraction and cDNA Cloning

The collected tissues were homogenized in liquid nitrogen, and total RNA were extracted using the TRizol reagent (Invitrogen, Carlsbad, California, USA) according to the manufacturer’s instructions. RNA integrity was confirmed by ethidium bromide gel staining and RNA quantity was determined spectrophotometrically at A_260_/_280_. Single-stranded cDNAs were synthesized by using PrimeScript™ One Step RT-PCR Kit (Takara, Dalian, China). Oligonucleotide primers (Table 1) were designed based on cDNA sequences of *N. vitripennis* KSPIs (GenBank accession numbers are NM_001161523 and NM_001170879). PCR conditions were as follows: an initial delay at 94 °C for 5 min; 34 cycles of denaturation at 94 °C for 30 min; annealing at 55 °C for 30s; and extending at 72 °C for 1 min. PCR products were fractionated in a 1% agarose gel by electrophoresis and purified with the DNA gel extraction kit (Aidlab, Beijing, China), and then cloned into the pGEM^®^-T easy cloning vector (Promega, Madison, Wisconsin, USA). Positive clones were selected by PCR and confirmed by sequencing at Invitrogen, Shanghai, China.

**Table 1:** Primers used in this study.

Primers Function	Primer Name	Primer Sequence（5ʹ-3ʹ）
**Gene cloning**	*NvKSPI-1*-F	TTGAGACGTGTCACCGAACA
	*NvKSPI -1*-R	ACTTGGCAATGGTAAGTTCTTG
	*NvKSPI -2*-F	ATGAAGTTAACCGTATTCCTCTTC
	*NvKSPI -2*-R	TTAAACAAGTCCGAAGTTTGCATC
**RT-qPCR**	RT-*NvKSPI-1*-F	ACTACCAACCAGTATGTGAC
	RT-*NvKSPI-1*-R	TCACTGTACTTGGCAATGGT
	RT-*NvKSPI-2*-F	CGGACGATTATGAAGAAGAAG
	RT-*NvKSPI-2*-R	ACTTGATAGCTTCCTCGTTAG
	RT-18S-F	TGGGCCGGTACGTTTACTTT
	RT-18S-R	CACCTCTAACGTCGCAATAC
**Recombinant expression**	*NvKSPI-1*-F-B	CGCGGATCCTGCATTTGTCCAAGAAACTACC
	*NvKSPI-1*-R-E	CCGGAATTCTTAACATTCACTGTACTTGGCAATG
	*NvKSPI-2*-F-B	CGCGGATCCCAACTTGAATCGGACGATTATGAAG
	*NvKSPI-2*-R-E	CCGGAATTCTTAAACAAGTCCGAAGTTTGCATCG

### 4.4. Sequence Analysis

The cDNAs and deduced amino acid sequences were analyzed by DNAStar software (version 5.02, DNAStar, Madison, Wisconsin, USA) and online Blast [52]. The signal peptides were analyzed by Signal P [53]. Multiple sequence alignments were carried out with DNAman software (Lynnon Biosoft, Quebec, QC, Canada) and Clustal W2 [54]. The phylogenetic tree was constructed by using MEGA 5.1 (Tokyo Metropolitan University, Tokyo, Japan) with the neighbor-joining (NJ) method.

### 4.5. Protein Expression, Purification, and Antibody Preparation

The primers (Table 1) containing *BamH I* and *Xho I* restriction enzyme sites were designed to amplify the ORF cDNA sequences of *NvKSPI-1* and *NvKSPI-2* by PCR. PCR products and the pGEX-4T-2 vector were ligated after they were double digested by *BamH I* and *Xho I* (Takara, Dalian, China). The recombinant plasmids were confirmed by sequencing, transformed into *Escherichia coli* BL21 (DE3) cells (AxyGen, Shanghai, China), and induced by 1 mM isopropyl thiogalactoside (IPTG) for protein expression. The recombinant proteins were analyzed by 12% SDS-PAGE and then purified using GST•Bind™ Resin Kit (Novagen, Hilden, Germany) according to the protocols. Protein concentrations were determined by using the BCA Protein Assay Kit (Novagen, Hilden, Germany).

### 4.6. Real-Time Quantitative PCR (RT-qPCR)

RT-qPCR was used to determine the transcription profiles of *NvKSPI-1* and *NvKSPI-2 in different tissues and ages of N. vitripennis* females. The 18S rRNA gene (accession number GQ410677) was used as an internal control. Ten microliters of each first-strand cDNA product were diluted with 90 µL of sterilized water before use. The RT-qPCR system was performed with each 20 µL reaction containing 10 μL SsoFast EvaGreen SuperMix (Bio-Rad, Hercules, California, USA), 1 µL forward primer (200 nM), 1 µL reverse primer (200 nM), 1 µL diluted cDNA, and 7 μL sterile water. The thermal cycling conditions were 95 °C for 30 s, followed by 40 cycles of 95 °C for 5 s and 60 °C for 34 s. Amplification was monitored on the iCycleriQ ^TM^ Real-Time PCR Detection System (Bio-Rad, Hercules, California, USA). The specificity of the SYBR-Green PCR signal was further confirmed by melting curve analysis. The experiments were repeated three times as independent biological replicates. The mRNA expression was quantified using the 2^−ΔΔC*t*^ method [55].

### 4.7. Serine Protease Inhibition Assays

Protease inhibition assays were performed with purified recombinant *NvKSPI-1* and *NvKSPI-2* proteins using the method described by Ling *et al*. [56]. Three typical serine proteases (bovine pancreatic trypsin, bovine pancreatic chymotrypsin, and proteinase K, 200 ng/mL) (Sigma, Taufkirchen, Germany) and their corresponding substrates (*N*-benzoyl-Val-Gly-Arg-*p*-nitroanilide, *N*-succinyl-Ala-Ala-Pro-Phe-*p*-nitroanilid, and *N*-succinyl-Ala-Ala-Pro-Phe-*p*-nitroanilid) (Sigma, Taufkirchen, Germany) were selected for determination of the spectrum of enzyme inhibition by recombinant *NvKSPI-1* and *NvKSPI-2* as follows. The recombinant *NvKSPI-1* and *NvKSPI-2* (1 µg each) were pre-incubated with a reaction buffer (100 mM Tris-HCl, 100 mM NaCl, 1 mM CaCl_2_, pH 7.5) containing 200 ng/mL serine protease for 30 min at room temperature, and then 200 μL substrate was added (0.1 mM, 100 mM Tris-HCl, 100 mM NaCl, 1 mM CaCl_2_, pH 7.5) before measuring the absorbance at 405 nm every min for 30 min. Substrates alone were used as blanks. Buffer and buffer with bovine serum albumin (BSA) were used as controls. One unit of enzyme activity was defined as an increase of absorbance by 0.001 per min. The experiments were repeated three times as independent biological replicates.

### 4.8. Prophenoloxidase (PPO) Activation and Phenoloxidase (PO) Activity Assays

PPO activation and PO activity assays were performed with purified recombinant *NvKSPI-1* and *NvKSPI-2* according to the method described by Ling *et al*. [48]. *M. domestica* in the white pupal stage were sterilized by 75% alcohol, rinsed with sterilized water and finally air-dried at room temperature before use. Hemolymph was collected from pupae by puncturing the pupal cuticle with a sterilized dissecting pin and rapidly transferred into a sterilized 1.5 mL Eppendorf tube. The collected hemolymph was then centrifuged at 3300 g for 5 min at 4 °C, and the supernatant was transferred into another 1.5 mL Eppendorf tube. Two microliters of each hemolymph sample were incubated with or without *Micrococcus luteus* (0.5 μg) in 10 μL of Tris buffered saline (TBS) at pH 7.4 in wells of a 96-well plate for 60 min at room temperature. Then 200 μL of l-dopamine (2 mM in TBS, pH 6.5) was added and absorbance at 470 nm was monitored. One unit of PO activity was defined as an increase of absorbance at 470 nm by 0.001 per min. Plasma samples with low PO activity when incubated alone but high PO activity after incubation with *M. luteus* were selected for subsequent PPO and PO activation assays.

For PPO activation assays, each 2 μL of prepared hemolymph was incubated with 10 μL TBS, 10 μL TBS/*M. luteus* (0.5 μg)/*NvKSPI-1* (1 μg) mixture, 10 μL TBS/*M. luteus* (0.5 μg)/*NvKSPI-2* (1 μg) mixture, 10 μL TBS/*M. luteus* (0.5 μg)/BSA (1 μg) mixture, 10 μL TBS/*M. luteus* (0.5 μg)/GST (1 μg) mixture, and 10 μL TBS/PTU (phenyl thiourea, saturated), respectively, for 20 min at 25 °C. Finally, 200 μL of l-dopamine substrate (2 mM) was added to each sample, and the PO activity was measured at 470 nm in a plate reader for 30 min. One unit of PO activity was defined as an increase of absorbance at 470 nm by 0.001 per minute.

For PO activity assays, each 2 μL of prepared hemolymph was incubated with 10 μL TBS/*M. luteus* (0.5 μg) mixture for 10 min at 25 °C. Then 2 μL TBS, 2 μL TBS, 2 μL *NvKSPI-1* (1 μg), 2 μL *NvKSPI-2* (1 μg), 2 μL BSA (1 μg), 2 μL GST (1 μg), and 2 μL PTU was added, respectively, and incubated for 10 min at 25 °C. Finally, 200 μL of l-dopamine substrate (2 mM) were added to each sample, and the PO activity was measured at 470 nm in a plate reader for 30 min. One unit of PO activity was defined as an increase of absorbance at 470 nm by 0.001 per min. All of the above experiments were repeated three times as independent biological replicates.

### 4.9. Statistical Analysis

All data were calculated as the mean ± standard deviation. Differences between samples were analyzed by one-way analysis of variance (ANOVA). Means were compared by least significant difference (LSD) tests. All statistical calculations were run using DPS software (version 8.01) [35] and statistical significance was set at *p* < 0.05.

## 5. Conclusions

In summary, we executed the molecular cloning and functional studies of *KSPI-1* and *KSPI-2* in *N. vitripennis*. We found that *KSPI-1* and *KSPI-2* are specifically expressed by *N. vitripennis* venom apparatus. The recombinant GST-*NvKSPI-1* and GST-*NvKSPI-2* can inhibit the PPO activation in host hemolymph and *NvKSPI-1* can inhibit the trypsin activity. Those findings suggest that *NvKSPI-1* and *NvKSPI-2* play a role in repressing host melaninization through inhibition of trypsin activity and PPO activation. However, more studies are needed to investigate the immune mechanism of KSPIs in insects.
